# Evaluation of nursing care delivery methods using in a university hospital: a qualitative study

**DOI:** 10.1590/1980-220X-REEUSP-2024-0254en

**Published:** 2025-02-21

**Authors:** Nermin Uyurdağ, Seyhan Çerçi, Satı Birbudak

**Affiliations:** 1 Dicle University Atatürk Health Services Vocational School, Diyarbakır, Turkey.; 2 Arel University Faculty of Health Sciences, Istanbul, Turkey.; 3 Dr. Siyami Ersek Thoracic and Cardiovascular Surgery Training and Research Hospital, Istanbul, Turkey.

**Keywords:** Nursing, Nursing care, Patient Care, Enfermagem, Cuidados de enfermagem, Assistência ao Paciente, Enfermería, Cuidados de Enfermería, Atención al paciente

## Abstract

**Objective:**

This study evaluates a university hospital’s nursing care delivery methods.

**Method:**

The population of the study, which was conducted using a qualitative research design and phenomenological approach, consisted of all charge nurses working in a state university hospital (n = 39). The sample was determined to reflect maximum diversity (n = 15). Data were collected through face-to-face interviews using a semi-structured interview form. Description, analysis, and content analysis were performed using the MAXQDA software package. Codes, sub-themes, and themes were created. Necessary permissions were obtained prior to the research.

**Results:**

The research data were grouped under five themes: the care delivery method used, the decision to use the care delivery method, the decision to use the care delivery method in case of disaster or crisis, the impact of the care delivery method on outcomes, and care delivery method training. A total of 13 sub-themes related to the themes were identified.

**Conclusion:**

It was found that there is no standard structure in the care delivery method, and nurses do not have training.

## RESUMO

**Objetivo:**

Este estudo avaliou os métodos de prestação de cuidados de enfermagem em um hospital universitário.

**Método:**

O estudo, realizado com base em um delineamento de pesquisa qualitativa e abordagem fenomenológica, teve como universo todas as enfermeiras responsáveis que trabalham em um hospital universitário público (n = 39). A amostra foi selecionada de forma a refletir a máxima diversidade (n = 15). Os dados foram coletados por meio de entrevistas presenciais utilizando um formulário de entrevista semiestruturado. A análise descritiva e de conteúdo foi realizada utilizando o pacote de software MAXQDA. Foram gerados códigos, subtemas e temas. As autorizações necessárias foram obtidas antes do início da pesquisa.

**Resultados:**

Os dados da pesquisa foram agrupados em cinco temas: o método de prestação de cuidados utilizado, a decisão de utilizar o método de prestação de cuidados, a decisão de utilizar o método de prestação de cuidados em situações de desastre ou crise, o impacto do método de prestação de cuidados nos resultados e a formação sobre o método de prestação de cuidados. Um total de 13 subtemas relacionados aos temas foi identificado.

**Conclusão:**

Constatou-se que não há uma estrutura padronizada no método de prestação de cuidados e que as enfermeiras não recebem treinamento a respeito.

## RESUMEN

**Objetivo:**

Este estudio evaluó los métodos de prestación de cuidados de enfermería en un hospital universitario.

**Método:**

El estudio, realizado utilizando un diseño de investigación cualitativo y un enfoque fenomenológico, incluyó a todas las enfermeras responsables que trabajan en un hospital universitario estatal como población del estudio (n = 39). La muestra se seleccionó para reflejar la máxima diversidad (n = 15). Los datos se recopilaron mediante entrevistas cara a cara utilizando un formulario de entrevista semiestructurada. El análisis descriptivo y de contenido se llevó a cabo utilizando el paquete de software MAXQDA. Se generaron códigos, subtemas y temas. Se obtuvieron los permisos necesarios antes de iniciar la investigación.

**Resultados:**

Los datos de la investigación se agruparon en cinco temas: el método de prestación de cuidados utilizado, la decisión de utilizar el método de prestación de cuidados, la decisión de utilizar el método de prestación de cuidados en situaciones de desastre o crisis, el impacto del método de prestación de cuidados en los resultados y la formación sobre el método de prestación de cuidados. Se identificaron un total de 13 subtemas relacionados con los temas.

**Conclusión:**

Se determinó que no existe una estructura estandarizada en el método de prestación de cuidados y que las enfermeras no reciben formación al respecto.

## INTRODUCTION

Nursing care is at the heart of health care and is essential to the delivery of health care services. The way in which care is provided is essential for effective nursing care, and this issue is the responsibility of the general management of the health care organization, especially the management of nursing services^(1)^. Healthcare workforce shortages, healthcare budget constraints, and quality and safety concerns have led to attempts to redesign the healthcare workforce; most of these initiatives have targeted nursing care delivery methods^(2)^. Many international organizations and institutions report the need to maintain nursing care in quality standards with professional nursing practices guided by the continuous improvement process^(3)^.

The nursing care delivery method defines a systematic approach to the organization of nursing care provided to patients^(4)^. These models explain how to organize and deliver care to recipients guided by values and beliefs^(5)^. In addition, the care delivery method as a concept is also expressed as meeting the needs of patients with independent or collaborative care approaches^(6,7)^. Care delivery models, shaped by nursing and management theories, involve the conceptual organization, coordination, and distribution of nursing services to caregivers^(8)^.

The Use of the care delivery method in patient care is to create a structure regarding how nurses directly involved in care are assigned to care work^(9)^. The main goal of successful and safe care in health care institutions where care services are provided is to provide the highest satisfaction, safety, and quality at the lowest cost. In order to achieve these goals, it is crucial to determine the most appropriate method of care delivery method that corresponds to the vision and mission of the organization^(10)^. The organization’s quality expectations related to the service, the philosophy, the cost of the care delivery method, the level of care required by the patient, the physical characteristics of the facility, and the number and quality of the nursing staff are critical to the choice of care delivery^(10)^. Because the nursing care delivery model is directly related to how care is planned, implemented, and organized, how patients are assigned to nurses, their competencies, and the expected outcomes^(11,12)^.

Institution management and executive nurses should take into account whether the institution supports the nursing services philosophy, whether the method has scientific validity, and whether it is suitable for organizational structures such as clinical decision-making, business department, communication, and management when determining the care delivery method to be applied^(1)^. In addition, executive nurses should consider all the differences in the planning, implementing, and evaluating care, from determining the patient’s care-related requirements to choosing the appropriate care delivery method^(10)^.

It is stated that choosing the appropriate nursing care delivery method has positive effects on ensuring high-quality standards, patient safety, patient and employee satisfaction, job satisfaction, and cost-effectiveness^(13)^. In addition, it contributes to improving the quality of care by increasing the commitment of nurses involved in care to work and their participation in decisions made^(14)^. Besides being a list of tasks, care delivery models enable nurses to choose what they want to do and comply with ethical rules^(15)^.

Two groups in the relevant literature examine nursing care delivery methods: classical and integrated methods. Classical methods include the case method (total patient care), functional nursing, team nursing, and primary nursing. Conceptually, functional nursing is considered a work-oriented method, while others are individual-oriented. It is stated that there is an open, human-oriented holistic approach to communication and interaction in individual-oriented methods^(13)^. The most commonly used integrated care delivery methods are case management, chronic care management, and integrated care improvement. Integrated care methods constitute the focal point of change in the health system, and it is stated that nurses have a significant role in implementing integrated methods due to their functions at all levels of the care process^(16)^.

This study aimed to evaluate the nursing care delivery methods used in a university hospital. For this purpose, answers were sought to the following questions.

1- What care delivery methods are used, and why do they prefer?
2- What impact do preferred care delivery methods have on outcomes?
3- What is the level of training nurses need regarding care delivery methods?

## METHOD

*Type of the research:* In this study, the descriptive phenomenological approach, one of the qualitative research methods, was adopted. Phenomenology reveals the participants’ experiences, shared meanings, and perceptions of the concept or phenomenon. This method is recommended to determine the factors related to the phenomenon and its effect on individuals^(17,18)^. This study used descriptive phenomenological qualitative design to examine ‘nursing care delivery methods’ as a phenomenon. The study data were reported using the COREQ checklist.

*The purpose of the research*: This study was conducted to evaluate the opinions of service-responsible nurses working in state university hospitals about nursing care delivery methods and the nursing care delivery methods they apply.

*The universe and sample of the research:* The population of the study was composed of responsible nurses working in clinics where patient care is provided at a public university hospital (N = 39). Although 15 responsible nurses were determined for the sample, it was aimed to expand the sample size to the number where the data reached satisfaction. The maximum diversity method, one of the purposeful sampling methods, was used to determine the sample^(19)^. For this purpose, three main groups were determined in which the patient care requirements, the number and quality of caregivers, nurse workload, and physical characteristics of the unit differed. In this direction, the research was conducted with 15 responsible nurses who agreed to participate voluntarily, including five working in intensive care units, five working in internal units, and five working in surgical units. Although the repetitions in the data started in the 14th interview, the research was terminated with the 15th interview to complete the sample. Since the participants’ statements were repeated in the interviews and no new information was obtained, the researchers decided that data saturation was achieved. No charge nurse refused to participate in the study.

*Data collection tool:* The research data were collected with a semi-structured interview form developed following the relevant field literature^(20,21)^. This form was created from questions questioning the introductory characteristics of the participants, their knowledge about nursing care delivery methods, whether they have received an education on this topic, whether education is required on this topic, the method of care delivery they use, the effects of these methods, the reasons for choosing the method, and the method of care delivery they use in the event of a disaster.

*Collection of data:* After explaining the scope and purpose of the research, the data were collected using a face-to-face, in-depth interview technique by making an appointment with the participants. The interviews were held between 15 June 2023 and 15 July 2023 in the units where the participants worked and in the offices of the nurses in charge. The interviews were recorded with a voice recorder with the participants’ permission. Each interview lasted an average of 35 minutes. The interviewer and other researchers have RN and PhD degrees in nursing. The interviewer and other researchers are experienced and trained in qualitative research. The interviews were conducted by a female researcher with the title of lecturer who has studies on care management. During the research process, the participants were contacted three times: making an appointment, interviewing, and sharing the interview transcript. Participants did not provide feedback after the interview transcript was shared.

*Evaluation of the data:* The audio recordings were decoded manually during the interviews. After the audio recordings of the interviews were transcribed, they were shared with the participants, and their approval was obtained again. The data analysis was done in three stages: description, analysis, and interpretation using content analysis^(19)^. MaxQDA 10 Plus program, a qualitative data analysis software, was used to code the research data. After the researchers had made the encodings separately, they were compared, and a consensus was reached on the codes considered different. The findings were interpreted by the created categories, themes, and sub-themes.

*Limitations of the research:* The results cannot be generalized since this research was conducted with responsible nurses working in state university faculty of medicine hospitals in a province in southeast Turkey.

*Ethical considerations:* Ethics committee approval was obtained from the Non-Interventional Clinical Research Ethics Committee of D University Faculty of Medicine. (12-04-2023 date/ decision number xx5) to realize the research. Application permission was obtained from the institution where the research will be conducted (12-06-2023 date/50xx23 decision number). The participants were informed about the research before the interview and obtained consent. In the statement, they were assured that their identities and the audio recording would remain confidential and not be used for any other purpose. The audio recordings were deleted after being deciphered. The codes determined by the researchers (the letter “P” and the interview sequence number) were used in the direct quotations included in the findings.

## RESULTS

Demographic findings from the interviews conducted in this research consist of information about nurses’ personal and professional characteristics such as gender, education, the unit they work in, profession, and years of work in the institution.

Of the responsible nurses participating in the research, five work in intensive care, five work in surgery, and five work as internal medicine nurses. It was determined that 80% of the participants were female, and 80% were undergraduate graduates. It was found that the average working year in the profession is 24 years (12–40), and the average working year in the institution is 13.5 years (5–28). The theme and sub- themes obtained from the individual in-depth interviews are in Table 1. When the results of in-depth interviews conducted with the responsible nurses about the functioning of the care delivery process are evaluated thematically, it is seen that they are grouped under five main themes: “applied care delivery method,” “deciding on the care delivery method,” “deciding on the care delivery method in case of disaster or crisis,” “the impact of the care delivery method on outcomes” and “care delivery method training.”

**Table 1:** Themes and sub-themes – Diyarbakir, Turkey, 2023.

	Main themes	Sub themes
Evaluation of Nursing Care Delivery Methods	Applied Care Delivery Method	Case Method
		Team Nursing
		Functional Nursing
		The Mixed Method
	Deciding on the Method of Care Delivery	Management‘s Request
		Number of Nurses
		The Patient‘s Need for Care
	Deciding on the Method of Care Delivery in Case of Disaster or Crisis	–
	The Effect of the Care Delivery Method on the Outputs	Reduction in Hospitalization Time
		Reduction of Infections
		Increasing Patient Satisfaction
		Increasing Employee Satisfaction
	Care Delivery Method Training	Getting An Education The Necessity of Education

**Figure 1:**
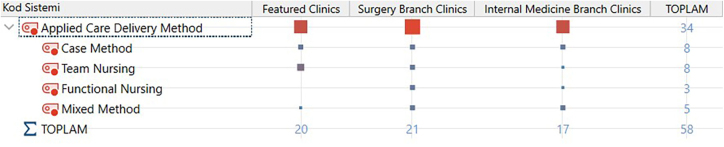
Applied maintenance delivery method theme and sub-themes.

*Findings Related to the Theme of Applied Care Delivery Method:* The opinions of the responsible nurses on the applied care delivery method theme were examined under four headings (case method, team nursing, functional nursing, and mixed method) (Figure 1).

*Case Method*: It was determined that the responsible nurses applied the total patient care (case method) method; morning seizure delivery was made to all nurses; sometimes, they shared patient rooms, and sometimes, they shared patients according to the patient’s care needs. Some references from the nurses’ opinions on the case method are found below:

*...the night team passes the shift to whoever comes in the morning. The whole team takes over all the patients, we share the patients. We try to make a fair distribution. Some of our patients may be in serious condition*... (P01).

*...of course, I give five patients to one nurse. I apply the case method. So one nurse is responsible for everything for 5 patients...* (P09).

‘...We share patients. We apply the system we call the case method... (P13).

*Team Nursing*: It was found that the charge nurses used methods in which more than one nurse worked as a group, and this method is called team nursing. Excerpts from the relevant opinions of the charge nurses are given below:

...*there is teamwork in intensive care. There is also teamwork in the clinic. As for patient sharing, since all nurses are experienced, for example, I have a new nurse right now, I gave her to my experienced nurse*... (P03).

...There are undergraduate graduates and associate degree graduates. There are those who have been working at the university for many years. We do not look at the difference in education when forming these teams. We look for people who get along with each other. Teams are reconstituted at the beginning of each month... (P08).

*Functional Nursing*: It was determined that responsible nurses employ the work-centered care delivery method. The references related to the sub-theme of functional nursing are given below:

...*each nurse will be responsible for one job. The advantage happens in every way. For example, it reduces hospital costs. For the patient, I think the length of stay in the hospital will be shorter*… (P02).

...*for example, in some clinics, there is a nurse who looks after only one job. For example, a nurse is just taking care of the dressing. The other is for medical treatments, the other one is preparing the patient at work, accepting the patient’s admission at work, and my other nurse is here, our extra procedures are taking place*... (P06).

*The Mixed Method*: In some cases, it was observed that more than one method was applied together in the delivery of patient care. Statements regarding this method, which is called mixed method by the researchers, are given below:

...*If there are 10 patients, they take care of 10 patients together. If there are 11 patients, 6 patients are transferred to one, 5 patients to another, and the patient who comes later is transferred to the one with 5 patients. And during the day, of course, they help each other in opening the vascular access and treatment. But when we primarily divide the patients, everyone takes care of their own patients*... (P06).

...Active case nursing is not realistic for us. Also, since there is a general shortage of nurses in our department, I use team nursing and assignment in a combined way in my clinic... (P14).

**Figure 2:**
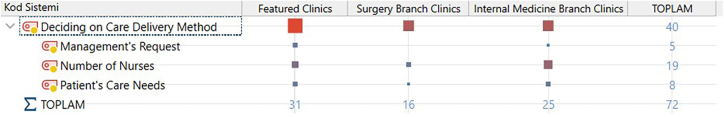
The theme of deciding on the care delivery method and its sub-themes.

*Findings Related to the Theme of Deciding on the Method of Care Delivery:* Nurses’ opinions on the care delivery method were examined under three headings (management request, number of nurses, and patient’s need for care) (Figure 2).

*Management Request*: Responsible nurses stated that they received support from the management in deciding on the care delivery method. Relevant quotes are included below:

... *Health care is a method that was jointly decided by our management, with our input. A method determined by the number of nurses and beds*... (P01).

…We tried sharing patients in the clinic. We tried sharing patient rooms with nurses. This system is valid since the number of nurses is low. Another reason why we prefer this method is that the senior management asked us to use the patient sharing method in quality audits.... (P13).

*Number of Nurses*: Responsible nurses also mentioned that the number of nurses is important in determining the care delivery method. They also emphasized that the number of nurses working in intensive care units is less than the number that should be required by legislation. Relevant excerpts are below:

...*We share patients according to the number of nurses. We work in such a way that every nurse knows every patient but provide care to her own patient*... (P01).

... While we need to give one nurse for every 2 beds, we share as 3 3 2 because there are 3 nurses working in intensive care in each shift.... (P05).

*Patient’s Care Need*: There are opinions stating that the patient’s need for care is also important in deciding on the method of care delivery. In this respect, there are statements that the workload should be shared equally. Related references are given below:

... *right now, most of our patients are already in need of care because they are over 50 years old. Do they take multiple antibiotics, if they have a lot of secretions, it is necessary to aspirate more often. Here, we can match this patient with a patient whose care is easier, and we are necessarily helping that nurse*... (P03).

*...I was evaluating according to whether the general condition of the patient was heavy or light, when I give the first two beds to a nurse friend or 3 beds to the first nurse friend, she knows what she is missing in her treatment. But when I don’t allocate patients, I may not be able to realize who gives how much adequate care to which patient or who gives incomplete care...* (P13).

*Findings Related to the Theme of Deciding on the Care Delivery Method in a Disaster or Crisis:* The sub-theme title could not be created from the opinions of the responsible nurses on the theme of the method of delivering care in disasters or crises. In such cases, there are opinions that they have not generally changed the care delivery method. The statements that they did not apply an extra maintenance presentation technique are below, mentioning that there were two earthquakes centered in Kahramanmaraş / Turkey on 06 February 2023 and that their province was significantly affected by these earthquakes:

...*we only received earthquake victims. Apart from our own nurses and staff, support nurses came. Of course, everyone still took care of all the patients. He provided all kinds of care services, but we did the same thing here*... (P11).

*...after the earthquake we recently experienced, our clinic became a crash clinic, all the patients who were under the cave-in in the earthquake and had crash syndrome were hospitalized in our ward, we worked on a patient basis at that time, but in a way that a nurse had at most 2 patients...* (P15).

**Figure 3:**
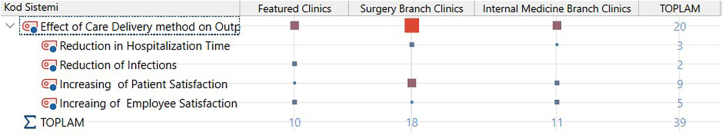
The theme and sub-themes of the impact of the care delivery method on outcomes.

*Findings on the Theme of the Impact of Care Delivery Method on Outcomes:* The theme of the effect of the care delivery method on outcomes was examined in four sub-themes: a decrease in hospitalization time, a decrease in infections, and an increase in patient and employee satisfaction (Figure 3).

*Reduction in Hospitalization Time*: Responsible nurses have stated that their care delivery method accelerates recovery and reduces hospitalization time. The references related to the subtheme of reduction in hospitalization time are given below:

...*we have 10 beds. When we do not distribute the patients to the nurses, when each nurse is responsible for these 10 patients, some things of the patients can be overlooked at that moment. But when a nurse takes his/her own patient, he/she will pay attention to the care and treatment of each patient and can make a better care plan and program for each patient, which affects the duration of the patient’s stay in the hospital, accelerates the recovery period, and reduces the cost*… (P08).

*...The patient’s length of stay in the hospital is also reduced because that person works very oriented because he/she takes care of the patient all day long, and because he/she knows everything very well because he/she does it properly, he/she hands over the patient in a very good way, so the infection is also reduced. Patient care time is also reduced. In fact, the patient is discharged very quickly...* (P10).

*Reduction of Infections*: Infection control is vital in the delivery of care. The responsible nurses reported that the presentation method they applied reduced the infection. The relevant citations are presented below:

...*When she was the only nurse, we chose this method regarding the infection risk. A single nurse is less contagious*... (P05).

... *Because of the high risk of infection, this way the risk of infection is minimized.*... (P07).

*Increasing Patient Satisfaction*: Nurses responsible for care management have stated that the method used increases patient satisfaction. The quotations related to the sub-theme of increasing patient satisfaction are given below:

...*It increases patient satisfaction a lot. As I said, the patient adopts the nurse. It also creates a positive energy. The nurse also adopts the patient. She knows her patient better*... (P04).

...*Increases patient satisfaction. Because the patient knows his nurse, he recognizes who is responsible for him during the day. They know their responsibilities better when each nurse has their own patients*... (K06).

*Increasing Employee Satisfaction*: Responsible nurses stated that the method they applied increases the satisfaction of nurses. The quotations related to the sub-theme of increasing employee satisfaction are given below.

... *What I observe from the employees is that they are satisfied with this situation. We already do this method in consultation with them*... (P02).

... *In this way, everyone has a good knowledge of the patients, all employees are familiar with all the patients, and we have seen that my colleagues are very satisfied. Because everyone works equally*... (P07).

**Figure 4:**
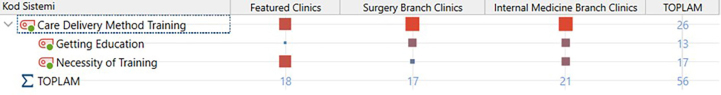
The theme of care delivery method training and its sub-themes.

*Findings Related to the Theme of Care Delivery Method Education:* The theme of care delivery method training was analyzed in two sub-themes as receiving training and necessity of training. (Figure 4).

*Getting An Education*: It was determined that charge nurses did not receive training on the care delivery method in general. The references related to the relevant theme are given below:

... *Our health services administration organizes in-service training on specific issues. However, we have not received a specific training covering the topic you are asking*... (P01).

...*We were not offered any special training on this subject. Apart from that, we were not given such a training in terms of very special certification*... (P09).

*The necessity of training*: Responsible nurses stated that there should be a training on care delivery methods:

... *It definitely should be. I think there should be, because this is a very important job. I think the nursing profession can be elevated to a higher level with this kind of training*... (P08).

... *Yes. I especially want it for clinical nurses, because we, the responsible nurses, are aware of many things because we attend most of the meetings. We come and inform the nurses, but how much we can do. That’s why I want these trainings especially for clinical nurses*... (P06).

## DISCUSSION

In this study, five main themes were reached: the applied care delivery method, deciding on the care delivery method, deciding on the care delivery method in case of disaster or crisis, the impact of the care delivery method on outcomes, and the care delivery method training.

The applied care delivery method is the first theme that was determined and consists of four subthemes. In this study, participants reported using case methods, team nursing, functional nursing, and mixed methods as care delivery methods. In a study conducted in units where acute patients are cared for in Australia, it was stated that the case method is widely used, similar to the findings of this study, and one of the most important reasons for this is staff shortages^(22)^. Another study also emphasized that the case management model is one of the oldest care delivery methods, and although it decreased after the 1930s, its reuse has become widespread today^(21)^. A study conducted in Portugal stated that the case method is a standard care delivery model. This preference is because following the same patient during a shift allows for more humane and personalized nursing care^(23)^. In this research, the combination of more than one nurse to serve together was called team nursing by the participants. “*I think the name fits team nursing, however. A different group of nurses comes every day. They share patients through existing patients and provide their care until the end of the day. A different group comes the next day. However, the people in the group do not change*”(P08). In the related field article, it is stated that a team can be mentioned for team nursing in the presence of a team leader and employees with different skills and education levels, and the care implementation model of this team is also defined as a team nursing model^(24)^. In their study, in which Arifin et al.^(25)^ compared the functional and team nursing models applied in a hospital, they reported that team nursing improves the quality of care, positively affects patient outcomes, and its application should be expanded outside primary care services. In another study, in addition to the positive and negative consequences of team nursing for nurses and the organization, it was reported that delegating authority between team members improved nursing care^(26)^. Another model applied by the participants is functional nursing. Although this method is not widely preferred due to its typical characteristics such as task orientation, fragmentation of care, and low patient satisfaction^(12,21)^, it is stated that there is an increase in its application due to increasing workload and bureaucratic reasons^(27)^. In this research, the combination of functional and case method models were described as mixed. A study mentions hybrid models in which multiple models are used together^(28)^. In light of this information, the findings of this research and the studies conducted on this subject are similar in terms of the care delivery methods used.

The second theme in the research is deciding on the care delivery method. The participants said they decided on the care delivery method they would apply according to the management’s request, the number of nurses, and the patient’s care needs. The related field article states that determining the care delivery method is one of the crucial responsibilities of nursing services managers and the general hospital manager. Deciphering the method, the institution’s objectives and possibilities, and the philosophy of nursing services are practical^(5)^. The financial resources of the institution and the insufficiency of the number and characteristics of the employees may cause the failure of the chosen method^(29)^. However, the nurse’s perception of her/his role as a professional determines her/his patient- or task-oriented approach^(5)^. A study mentions the positive results of providing care services by a team of nurses with different levels of competence in meeting the patient’s care needs^(30)^. In this study, the participants stated that the number of nurses was insufficient. “*The number of nurses is insufficient in intensive care conditions. However, at least they can take it as a collaboration. We also have an insufficient number of nurses in the clinic. We have two nurses working in 20 beds. It is also on duty during the day*”(P13). However, due to the preference of case method and functional nursing as the care delivery method, the number of employees is decisive in deciding on the care delivery model.

The third theme is the method of providing care in the event of a disaster or crisis. Participants reported that they did not change the method of care they used in a disaster or crisis. One study on this topic reported that there is no definite process, way, or approach for which care delivery method is chosen in which situation. In addition, it is stated that the needs required by the patient’s condition and how the care is conceptualized are also important^(5)^. Prentice et al.^(31)^ investigated the effect of care delivery models on professional collaboration and reported that care delivery models should be adaptable according to emerging situations. Another study mentioned the positive outcomes of team care provided to staff with different professional qualifications during the pandemic^(26)^. The participants, whose work intensity increased due to the February Six Earthquakes, noted that they managed this crisis by increasing the number of nurses in the management. “*Yes, direct care plans were developed immediately. Again, since we cannot provide care on a per-patient basis, a support team came to us from outside clinics. We have again actively maintained our system”*(P14).

The effect of the care delivery method on outcomes is the fourth theme of the research. A decrease in hospitalization time, a decrease in infections, an increase in patient satisfaction, and an increase in employee satisfaction are the sub-themes identified under this theme. A study investigating the effect of nursing care delivery models on quality and safe care stated that team nursing, especially where cooperation is high, has a positive effect on the safety and quality of care^(4)^. A study that investigated the effect of care delivery models and emotional exhaustion on the quality of care found that the quality of care was affected by emotional exhaustion rather than the presentation model^(32)^. Winslow et al.^(33)^ stated that the mixed model they examined in their study increased nurse satisfaction. However, it did not affect patient outcomes such as falls, pressure injuries, or patient satisfaction. At the same time, King et al.^(34)^ reported that both methods had no significant effect on nurses’ job satisfaction, stress level, and turnover rate in their study, in which they compared the case method and team nursing. While Havaei et al.^(4)^ stated that nurses working with the team nursing method reported more incomplete care than nurses working with the case method, in another study where the results of the transition from case method to team nursing were examined, it was found that there was no increase in nurses’ job satisfaction. However, care was perceived more effective with team nursing^(35)^. Fernandez et al.^(36)^ reported that the nursing team effectively reduced medication errors and adverse intravenous outcomes, decreasing pain scores, but did not change the fall incidence of patients. Since it is stated that the care delivery methods used by the participants in this study have positive effects on patient and employee outcomes, these results are partially compatible with the results of the studies conducted on this subject.

The fifth theme identified in this research is the issue of training in care delivery methods. The participants stated that they did not receive any training on care delivery methods, but that training on this issue is necessary. The relevant literature emphasizes that nurse managers should follow scientific articles on this topic when deciding which care delivery method to use or when changing an existing method, and evaluate the impact of the method on facility, nurse, and patient outcomes^(1)^. From this perspective, knowledge of all aspects of care delivery methods becomes important in determining the most appropriate method for nurse leaders. However, nursing care delivery methods are not included in the scope of continuing education and certificate programs set by the Ministry of Health in Turkey^(37)^. This topic is included in the content of nursing management courses in undergraduate nursing education^(38)^. The participants did not receive undergraduate education in this subject because their graduation dates were before the date of inclusion of this subject in undergraduate education.

## CONCLUSION

The study’s findings indicate a preference for classical methods in care delivery, with the number of employees serving as a primary factor in method selection. The prevailing method is maintained in the event of a disaster or crisis. It is hypothesized that the selection of an appropriate care delivery method has a positive impact on outcomes. However, there is a perceived lack of training for responsible nurses in this area. The necessity of training in this subject is acknowledged. In alignment with these findings, it is recommended that health organizations, particularly those with nursing management, be equipped with the necessary resources to implement the most suitable care delivery methods and to establish training programs in this regard, with the aim of enhancing the quality of nursing care.
